# Characteristics of Recurrent Hepatocellular Carcinoma Based on Serum AFP, PIVKA-II, and Genetic Mutations

**DOI:** 10.3390/medicina62030508

**Published:** 2026-03-10

**Authors:** In Soo Cho, Keun Soo Ahn, Sangkyun Jeong, Tae-Seok Kim, Min Jae Kim, Seung Kyoung Yang, Sunwha Cho, Yong Hoon Kim

**Affiliations:** 1 Division of Hepatobiliary and Pancreatic Surgery, Department of Surgery, Keimyung University Dongsan Hospital, Daegu 42601, Republic of Korea; brianchokr@naver.com (I.S.C.); mjk2976@gmail.com (M.J.K.);; 2 Institution for Cancer Research, Keimyung University Dongsan Medical Center, Daegu 42601, Republic of Korea; 3 Keyomics Co., Ltd., Daejon 34013, Republic of Korea

**Keywords:** carcinoma, hepatocellular, biomarker, mutation, alpha-fetoproteins, acarboxyprothrombin

## Abstract

*Background and Objectives:* Reliable tools for evaluating tumor biology and forecasting clinical outcomes in recurrent hepatocellular carcinoma (HCC) remain scarce, and molecular characterization through genetic profiling is equally limited in this setting. This investigation explores whether serum tumor marker expression patterns correlate with genomic mutation profiles, and whether such correlations may facilitate more accurate prediction of tumor biology and patient prognosis in recurrent HCC. *Materials and Methods:* We analyzed a cohort of 20 patients who underwent curative-intent resection for both primary and recurrent HCC. Tumor specimens collected at the time of each operation were subjected to targeted next-generation sequencing for mutation profiling. Based on pre-operative serum levels of AFP (alpha-fetoprotein) and PIVKA-II (Protein Induced by Vitamin K Absence or Antagonist-II) measured before each surgery, patients were stratified into four biomarker subgroups. Those who maintained the same biomarker subgroup at both operations were designated the ‘serum concordant group’, whereas those who transitioned between subgroups were classified as the ‘serum discordant group’. Clinical characteristics and mutation data were subsequently compared between these two classifications. *Results:* The interval from primary surgery to disease recurrence was significantly shorter in the serum concordant group relative to the serum discordant group (mean 11.16 ± 1.86 vs. 44.8 ± 9.45 months, *p* < 0.001). Additionally, disease-free survival following reoperation was significantly inferior in the concordant group compared with the discordant group (*p* = 0.039). Regarding mutational patterns, the concordant group demonstrated shared gene mutations between primary and recurrent lesions, while the discordant group exhibited divergent mutational landscapes across both timepoints. *Conclusions:* The concordance or discordance of serum tumor marker profiles between primary and recurrent HCC lesions may serve as a clinically accessible surrogate for underlying tumor biology and prognostic stratification. These results are preliminary and hypothesis-generating. Further studies in larger, independent cohorts are warranted to confirm the observed associations.

## 1. Introduction

Definitive diagnosis of hepatocellular carcinoma (HCC) relies primarily on cross-sectional imaging modalities such as computed tomography (CT) and magnetic resonance imaging (MRI), while serum biomarkers—alpha-fetoprotein (AFP) and protein induced by vitamin K absence or antagonist-II (PIVKA-II, also referred to as des-gamma-carboxyprothrombin [DCP])—serve complementary roles in disease surveillance and prognostic assessment. Recent reviews highlight advances in noninvasive tools integrating imaging with serum and liquid biopsy biomarkers (e.g., circulating tumor cells); however, translation into routine clinical practice remains limited [1,2]. When used individually, each biomarker demonstrates suboptimal sensitivity across the full spectrum of HCC stages. Nevertheless, combining AFP and PIVKA-II enhances both diagnostic sensitivity and prognostic accuracy [3,4]. Given the molecular and phenotypic heterogeneity of HCC (including diverse driver alterations and pathway activation), the number of elevated tumor markers varies among tumors. Approximately one-fifth of HCC patients have concurrently elevated AFP and PIVKA-II, another 40% show elevation of only one marker, and the remaining fifth exhibit normal values for both [5,6]. Evidence suggests that this heterogeneity in biomarker status reflects underlying differences in clinicopathological tumor characteristics [7,8,9].

Significant advances in genomic technologies have fueled extensive efforts to characterize the genetic drivers of HCC development and progression. Recurrently altered genes—including TERT promoter, TP53, and CTNNB1—alongside pathway dysregulation involving EGFR, TGFB, VEGF, and receptor tyrosine kinase signaling have been implicated as central contributors to hepatocarcinogenesis [10,11]. These mutational differences delineate distinct molecular subtypes with differing prognostic implications [10,12,13]. Our group previously demonstrated that AFP and PIVKA-II expression levels are linked to specific genomic profiles, raising the possibility that tumor genetic features might be inferred noninvasively from routine blood-based measurements [6].

Following initial treatment, HCC recurs at high rates, and recurrent disease can be managed using approaches analogous to those applied to primary tumors [14]. Intrahepatic metastasis (IM), which shares similar biology with primary cancer [15,16], is associated with poor prognosis. In contrast, multicentric occurrence (MO), representing de novo carcinogenesis at a distinct hepatic site, generally carries a more favorable prognosis owing to its independent biological origin. Elucidating the molecular basis of recurrence is therefore critical for tailoring subsequent therapeutic strategies. In practice, however, comprehensive genetic testing at the time of recurrence is not universally feasible due to cost and logistical constraints, and no validated clinical algorithm currently distinguishes IM from MO without tissue-based analysis [14,17]. Furthermore, our understanding of how serum tumor markers relate to the molecular biology linking primary and recurrent tumors remains limited.

We hypothesized that the stability of AFP/PIVKA-II phenotypes between primary and recurrent tumors could mirror underlying clonal relatedness. If the expression patterns of AFP and PIVKA-II are similar between the primary and the recurrent mass, the recurrent mass is likely to be an intrahepatic metastasis with a molecular biology similar to that of the primary mass. On the other hand, if the expression patterns of AFP and PIVKA-II differ between the primary and recurrent masses, it is possible that the recurrent mass represents a multicentric occurrence with a molecular biology distinct from that of the primary mass. To evaluate this hypothesis, we performed a paired analysis correlating serum biomarker concordance with genomic mutation profiles in a cohort of patients undergoing resection for both primary and recurrent HCC.

## 2. Materials and Methods

### 2.1. Study Population

This retrospective study included 20 patients treated at a single institution who underwent a second hepatic resection for recurrent HCC between 2011 and 2021, following prior curative resection of primary HCC. Eligibility required the availability of cryopreserved tumor tissue from both operations for genomic analysis. Serum AFP and PIVKA-II were measured preoperatively at each surgical episode, and DNA extracted from both primary and recurrent tumor specimens along with matched adjacent non-neoplastic liver tissues were subjected to next-generation sequencing.

### 2.2. Group Discrimination for Analysis

Patients were categorized into 4 groups based on the number of elevated tumor markers measured before the first and second surgeries. Patients were classified into one of four biomarker subgroups at each time point according to which markers were elevated at the time of surgery: Group A (AFP elevation only), Group B (PIVKA-II elevation only), Group C (both markers elevated), and Group D (neither marker elevated Elevation was defined using established thresholds of 20 ng/mL for AFP and 40 mAU/mL for PIVKA-II (equivalent to 7.5 ng/mL DCP), consistent with our prior publication [6]. Patients whose biomarker subgroup remained unchanged between the primary and recurrent operations were assigned to the “Serum concordant group”, while those who transitioned to a different subgroup were classified as the “Serum discordant group”. Among the 21 genes in which mutations were detected, genetic concordance between primary and recurrent tumors was re-evaluated by selecting genes relevant to hepatocarcinogenesis. The primary analysis focused on established HCC driver genes (TP53, PIK3CA, PTEN, and TSC2) [10]. In addition, a secondary exploratory analysis included low-frequency, context-dependent genes involved in DNA damage repair or cell adhesion (PALB2, CDH1, and MUTYH) [18,19,20]. Genetic concordance was defined as the presence of at least one identical mutation in these selected genes between primary and recurrent tumors. If patients had different genetic mutations in the primary and recurrent masses, they were included in the “Genetic discordant group”.

### 2.3. Fresh Tissue Samples Storage

HCC tissues with surrounding normal liver tissues as paired control were available in all patients. Fresh tissue samples of primary tumor and matched surrounding normal liver tissues were surgically obtained and saved in the frozen status (−80 °C). A pathologist examined all hematoxylin–eosin-stained slides to confirm HCC. Genetic analyses were performed using frozen tumor tissue, and the diagnosis of hepatocellular carcinoma was histologically confirmed after surgical resection.

### 2.4. Adaptor Preparation for Suppression PCR

Genomic alterations in the cancer genome were analyzed using a targeted precision sequencing method called Strandwise and Molecularly indexed Landmark Enrichment sequencing. This approach employs suppression PCR for multiplex target enrichment and incorporates unique molecular identifiers and strand identifiers to achieve ultra-high sequencing. A 71-nucleotide-long oligonucleotide (5′-AGGACCGTGTGCTGACACTCTTTCCCTACACGACGCTCTTCCGATCTNNNNNNNNCAGCTGACGTCAGTCT-3′) containing a molecular index of eight random nucleotides was hybridized to a shorter, 5′-phosphorylated oligonucleotide (Pi-GACTGACGGCAGCTG-3′) to create the SupL5 adaptor, which features a partial duplex region with a single mismatched base pair next to the molecular index. Hybridization was conducted by heating the oligonucleotide pair to 95 °C for 5 min, followed by stepwise cooling to room temperature at a rate of 1 °C per minute. Subsequently, the partial duplex DNA underwent a polymerization reaction to fill the single-stranded region, including the molecular index, using Klenow Fragment (3′→5′ exo-) (NEB, Ipswich, MA, USA) at 37 °C for 20 min. The SupL5 adaptor was then prepared by purifying the fully duplexed DNA, except for the 3′ T overhang and mismatch, using ExpinTM PCR SV (GeneAll, Seoul, Republic of Korea), and dissolving it in elution buffer to achieve a concentration of 16 µM.

### 2.5. Primer Preparation

Primers were designed to optimize the enrichment of the coding regions of the target genes. A list of primer candidates was generated using Primer3 (version 4.1.0) [21] within the 400 bp regions adjacent to the BstNI site or within regions flanked by BstNI sites, encompassing the coding sequences of the target genes. Two primers with identical orientation were chosen for each target coding region for nested suppression PCR based on the following criteria: (1) primer binding sites in the reference genome should not exceed three when allowing for a single-base mismatch, (2) primers covering larger coding regions were prioritized, and (3) the calculated melting temperature should be no more than 3 °C away from the average. The primers were organized into four groups according to their use (first or nested PCR) and their orientation with respect to the reference genome sequence: first forward, first reverse, nested forward, and nested reverse. In total, 2724 primer pairs were synthesized for 167 cancer-related genes.

### 2.6. Library Preparation for Precision Targeted Sequencing

One hundred nanograms of genomic DNA was digested with 10 units of BstNI restriction enzyme (NEB, Ipswich, MA, USA) at 60 °C for 4 h. The fragmented DNA was then purified using ExpinTM PCR SV (GeneAll, Seoul, Republic of Korea) according to the manufacturer’s instructions and eluted in 30 µL of elution buffer. The purified DNA underwent end-repair and A-tailing in a reaction containing dNTP and SolgTM Taq polymerase (Solgent, Daejeon, Republic of Korea) at 65 °C for 30 min, which was then purified using DNA Clean & ConcentratorTM-5 (Zymo Research, Irvine, CA, USA) following the manufacturer’s instructions and eluted in 6 µL of elution buffer. The entire volume of the 3’A-tailed DNA was mixed with 1 µL of 16 µM SupL5 adaptor and 7 µL Blunt/TA ligation Master Mix (NEB, Ipswich, MA, USA), and incubated at room temperature for 20 min. This ligation reaction was subsequently purified again using ExpinTM PCR SV and eluted in 40 µL of elution buffer. A measure of 2 µL of the adaptor-ligated DNA served as the template for the nested PCR reaction, which utilized two sets of primer pairs: one specific for the target sequences and the other for the adaptor sequence. The first PCR reaction was conducted using SolgTM h-Taq polymerase, initiating with polymerase activation at 95 °C for 15 min followed by 20 cycles of 95 °C for 10 s, 58 °C for 30 s, and 68 °C for 1 min The second nested PCR used 1 μL of the product from the first PCR, with polymerase activation at 95 °C for 15 min followed by 10 cycles of 95 °C for 10 s, 58 °C for 30 s, and 68 °C for 1 min. Finally, an aliquot of the Nested PCR product underwent an additional 10 cycles of PCR using sample index primers to add a sample index and to form the sequencing library structure for the Illumina platform. The sequencing was performed using Illumina HiSeq X Ten (Illumina, Inc., San Diego, CA, USA).

### 2.7. Sequence Analysis

Sequences of paired-end reads, structured with a molecular index, short duplex sequence with strand mark, and one target sequence in the 5′ to 3′ order, were selected. The details regarding the library name, read identifier, and the combination of molecular index and strand mark were stored separately. Among the unique sequences collected, those appearing more than twice across all libraries were aligned to the human reference genome, GRCh38/hg38, using the following BLAST+(v2.13.0) command and options: blastn-query QUERYFILE-db REFERENC -perc_identity 95-max_target_seqs 5-max_hsps 5-outfmt “6 qseqid sseqid pident length mismatch qstart qend sstart send sstrand btop evalue bitscore” -out OUTFILE. The resulting BLAST output was analyzed, and sequences were mapped to the locus with the highest bit score. If multiple loci shared the same bit score, a representative locus was selected based on the earliest chromosome number and position. Using these mapping results, the reads from each library were organized by their molecular index and original strand at reference loci to construct a single-strand consensus, followed by a double-strand consensus for those indices. Effective double-strand consensus sequences were those built from two single-strand sequences, each supported by at least two reads. For each molecular index, double-strand consensus sequences were compiled for each mapped locus from heptaplicated libraries, and a list of variant sequences was created. Positions with discrepancies between the two strands were deemed ineffective. Variants located within a single nucleotide homopolymer of more than 10 base pairs were also considered ineffective due to frequent indels from sequencing errors. A list of targeted genes is provided in the Supplementary Materials Table S1.

### 2.8. Statistical Analysis

All statistical analyses were conducted using IBM SPSS Statistics, version 25.0 (IBM Corp., Armonk, NY, USA). Continuous variables were assessed for normality. Normally distributed variables are presented as mean ± SD and compared using Student’s *t*-test, whereas non-normally distributed variables (including AFP and PIVKA-II) are presented as median (IQR/range) and compared using the Mann–Whitney U test. Categorical variables were analyzed with the chi-squared test or Fisher’s exact test, as dictated by expected cell frequencies. Recurrence-free and overall survival rates were estimated using the Kaplan–Meier method, and between-group differences were assessed by the log-rank test. This work was based on the author’s (In Soo Cho) doctoral dissertation completed at Keimyung University [22].

## 3. Results

### 3.1. Patient Characteristics and Clinical Outcome

**Figure 1:**
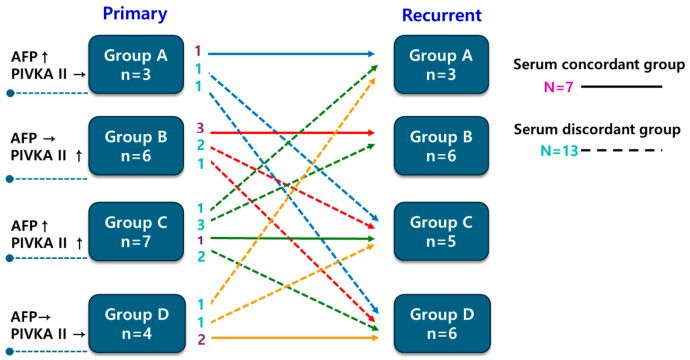
Serum tumor marker patterns in primary and recurrent tumors.

The study cohort comprised 20 patients (19 men and 1 woman) with a median age of 57 years. Hepatitis B virus infection was the predominant underlying liver disease (*n* = 15; 75%). The median duration of follow-up after the first operation was 78.64 months (range, 6.27–204.73 months), and the 5-year overall survival rate was 83%. Based on changes in biomarker subgroup assignment between the two operations, 7 patients met criteria for the serum concordant group and 13 for the serum discordant group. The distribution of primary tumor marker subgroups did not significantly predict whether recurrent tumors would be classified as concordant or discordant (Figure 1, *p* = 0.510).

### 3.2. Clinical Outcome Based on Serum Biomarkers Between Primary and Recurrent HCC

**Figure 2:**
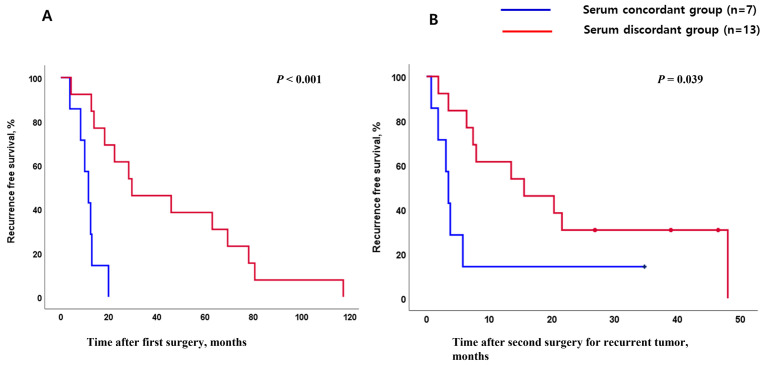
Survival analysis based on serum tumor marker patterns: (**A**) disease-free survival after the initial operation, until the first recurrence (before the second operation); (**B**) disease-free survival after the second operation, until the second recurrence.

Baseline characteristics were well-balanced between the two groups (Table 1). The time elapsed between the first operation and radiologically confirmed disease recurrence was markedly shorter among patients in the serum concordant group compared with those in the discordant group (Median 11.4 months vs. 29.5 months, *p* < 0.001, Figure 2A). Beyond this difference in recurrence timing, the serum concordant group demonstrated significantly abbreviated disease-free survival following the second resection relative to the discordant group (*p* = 0.039, Figure 2B).

### 3.3. Association Between Serum Biomarker and Genetic Mutation Profile

**Figure 3:**
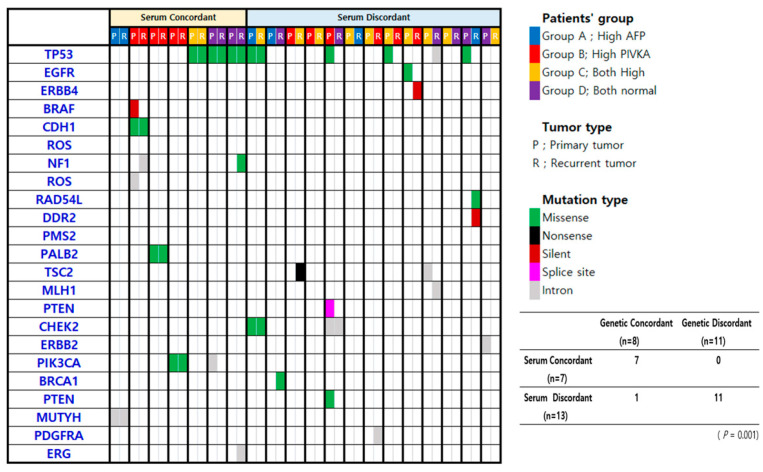
Association between serum biomarker expression pattern and genetic mutation in primary and recurrent HCC.

Targeted next-generation sequencing was successfully performed in all 20 patients. Pathogenic or likely pathogenic mutations were identified in 19 individuals, encompassing 42 mutation events distributed across 21 genes; TP53 was the most frequently mutated gene (*n* = 9, 32.14%). One patient had no detectable somatic mutations. All 7 patients in the serum concordant group also fell within the genetic concordant group, meaning their primary and recurrent tumors shared at least one identical mutation in the evaluated genes. Among the 13 patients in the serum discordant group, 11 were classified as genetically discordant—having no shared mutations between their two tumors. A strong statistical association was observed between serum biomarker concordance and genetic concordance (*p* = 0.001). Mutation landscapes in primary and recurrent tumor pairs, organized according to serum concordance status, are summarized in Figure 3.

## 4. Discussion

HCC typically develops in the context of chronic hepatic inflammation and fibrosis arising from viral hepatitis, chronic alcohol use, or metabolic-associated steatotic liver disease. This cirrhotic condition creates a permissive environment for multistep hepatocarcinogenesis and substantially elevates the risk of intra-hepatic disease recurrence following curative treatment. Recurrent lesions are biologically heterogeneous: some represent aggressive intrahepatic spread of the original tumor, while others arise independently and may carry a more favorable prognosis [23,24]. Developing individualized treatment algorithms for recurrent HCC demands accurate characterization of tumor biology; however, tools capable of reliably predicting the nature of recurrence at a clinical level remain inadequate.

**Figure 4:**
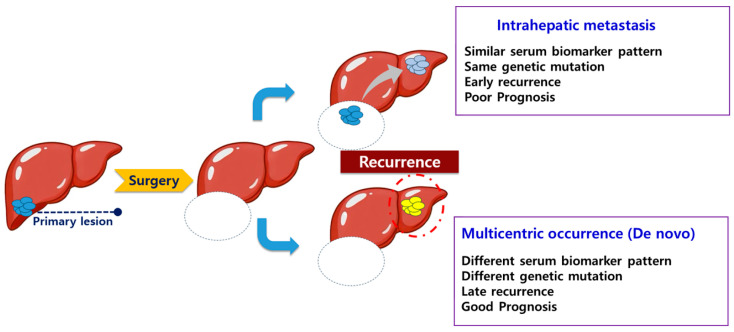
Schematics of the hypothesis.

The principal finding of this study is that the pattern of serum tumor marker expression—whether AFP and PIVKA-II profiles remain concordant or shift discordantly between primary and recurrent tumors—is significantly correlated with underlying genomic mutation concordance and with recurrence-free survival outcomes. (Figure 4) Patients whose biomarker subgroup was preserved at recurrence harbored tumors with shared mutational features, consistent with intrahepatic metastasis, and experienced both earlier recurrence and inferior post-reoperative outcomes. This association implies that serum biomarker patterning is not merely an epiphenomenon but reflects the clonal relationship between synchronous or metachronous tumors, offering a potential noninvasive window into the tumor’s molecular architecture.

Even in recurrent HCC, MO and IM exhibit distinctly different patterns. IM reflects intrahepatic spread of the original tumor and is linked to more aggressive disease and poorer survival, whereas MO indicates new, independent primary tumors in the liver and is generally associated with better overall and disease-free survival [14,17,25]. The ability to reliably discriminate between these two entities would have meaningful implications for treatment intensity and candidacy for aggressive local therapies, including re-resection or liver transplantation. Molecular approaches based on comparative mutation profiling have been evaluated for this purpose and offer mechanistic precision; however, their implementation in clinical practice is constrained by both cost and the time required for results [26,27,28,29].

Our findings support the concept that concordantly expressing patients—those whose biomarker subgroup remained stable—experienced earlier disease return after initial resection and derived diminished benefit from reoperation, consistent with an IM pattern of biologically aggressive, clonally related disease. In contrast, patients with discordant biomarker shifts demonstrated genetically distinct tumor pairs and favorable post-recurrence trajectories, in line with MO. These observations collectively suggest that serum biomarkers function as an accessible indirect index of the molecular relationship between sequential HCC lesions, and could potentially guide clinical decision making—for example, by identifying patients with concordant profiles who might benefit most from liver transplantation evaluation or from bridging systemic therapies prior to any planned resection.

Approximately 20% of HCC patients maintain normal levels of both AFP and PIVKA-II, which has traditionally been considered a limitation of serum biomarker–based approaches. However, our results indicate that this subgroup represents a distinct serum biomarker expression pattern with unique clinical and genetic characteristics. In line with previous studies, Group D has been associated with specific molecular alterations, and favorable clinical outcomes [6]. Moreover, serum concordance or discordance between primary and recurrent tumors within this group closely reflected genetic concordance, underscoring the biological relevance of normal serum biomarker profiles.

For patients with unresectable or metastatic HCC, systemic therapy constitutes the backbone of management. Tyrosine kinase inhibitors such as Sorafenib—as well as emerging agents including Linifanib and Erlotinib—are employed in this population, though response rates are variable due in part to the extensive molecular heterogeneity of HCC [30]. Predicting which tumor subtype will respond to a given systemic agent remains an unmet clinical challenge, and the proportion of patients deriving meaningful benefit from targeted therapy is currently modest [31]. Our data raise the possibility that biomarker concordance profiling could provide preliminary guidance on likely mutation profiles, potentially informing eligibility for and the choice of systemic regimens.

This study has several limitations. Given the small sample size and retrospective single-center design, our findings should be regarded as preliminary and hypothesis-generating rather than confirmatory. Detailed comparisons across biomarker subgroups were constrained by the overall small cohort size. In Group D patients, for instance, concordant normal biomarker profiles might still reflect favorable tumor biology, yet the small number of cases in this subgroup made any such inference unreliable. The overall limited sample size further prevented rigorous validation of the study’s key findings. Our cohort was markedly male-predominant (19/20), limiting generalizability and precluding sex-stratified analyses. Although NGS was used, the targeted sequencing panel did not capture all possible driver mutations, underscoring the need for confirmation in larger cohorts. Furthermore, serum tumor marker patterns may vary with tumor stage at detection, as marker levels tend to rise with cancer progression. In advanced HCC, therefore, serum biomarkers may be less accurate in reflecting the underlying genetic profile. Although CTNNB1 mutations were not detected in this cohort, this may be attributable to the limited sample size and the small proportion of patients with normal AFP and PIVKA-II levels, a subgroup in which CTNNB1 mutations are known to be most prevalent. Larger studies with balanced biomarker-defined subgroups are warranted to better capture the molecular heterogeneity of HCC.

## 5. Conclusions

This study demonstrates that the expression patterns of serum tumor markers—AFP and PIVKA-II—between primary and recurrent HCC are significantly associated with the genetic mutation profiles and clinical outcomes of recurrent tumors. The findings highlight the potential clinical utility of simple, noninvasive serum biomarkers as surrogate indicators of tumor biology in recurrent HCC. Given the challenges and costs associated with comprehensive genetic profiling, serum marker patterns could serve as a practical tool for stratifying patients, guiding personalized treatment strategies, and informing prognosis. Further large-scale, prospective studies are warranted to validate these findings and integrate biomarker-based approaches into routine clinical decision making for recurrent HCC.

## Figures and Tables

**Table 1:** Patient Characteristics.

Characteristic (Tumor Marker)	Serum Concordant (*n* = 7)	Serum Discordant (*n* = 13)	*p*-Value
Sex, *n* (%). Female/Male	0/7 (100)	1 (7.7)/12 (92.3)	0.452
Age, mean (SD ^a^)	59.1 (10.32)	56.85 (6.20)	0.444
Body mass index, mean (SD), kg/m^2^	24.42 (3.37)	24.41 (1.93)	0.994
1st AFP ^b^ (ng/mL) (median, range)	12.4 (2.19–7069.10)	26.8 (1.06–22,609)	0.350
2nd AFP (ng/mL) (median, range)	5.3 (1.3–10,684)	23.7 (2.2–18,133.10)	0.350
1st PIVKA-II ^c^ (mAU/mL/mL) (median, range)	54 (26.74–509.3)	150.6 (24–4302)	0.350
2nd PIVKA-II (mAU/mL/mL) (median, range)	44 (16.7–6881)	68.1 (19.87–8656)	1.000
Risk factors, *n* (%)			0.438
Hepatitis B	4 (57.1)	11 (84.6)	
Hepatitis C	1 (14.3)	1 (7.7)	
Alcohol	1 (14.3)	1 (7.7)	
Fatty liver (MASLD ^d^)	1 (14.3)	0	
Tumor size (Primary, median, range)	3.7 (1.3–6)	3 (1.7–9)	1.000
Vascular invasion (Primary), *n* (%)	2 (28.6)	3 (23.1)	0.787
Tumor size (Recurrent, median, range)	3 (1.1–16.7)	2.5 (1.5–7)	0.642
Vascular invasion (Recurrent) *n* (%)	2 (28.6)	4 (30.8)	0.919

^a^ SD: Standard Deviation; ^b^ AF: Alpha-fetoprotein; ^c^ PIVKA-II: Protein Induced by Vitamin K Absence or Antagonist-II; ^d^ MASLD: Metabolic dysfunction–Associated Steatotic Liver Disease.

## Data Availability

Data are available from the corresponding author upon reasonable request.

## Supplementary Materials

The following supporting information can be downloaded at: https://www.mdpi.com/article/10.3390/medicina62030508/s1, Table S1: List of targeted genes.

## Abbreviations

The following abbreviations are used in this manuscript.

HCC	Hepatocellular Carcinoma
AFP	Alpha-Fetoprotein
PIVKA-II	Protein Induced by Vitamin K Antagonist-II
CT	Computed Tomography
MRI	Magnetic Resonance Imaging
IM	Intrahepatic Metastasis
MO	Multicentric Occurrence
