# Supplementary material for: Characteristics of Recurrent Hepatocellular Carcinoma Based on Serum AFP, PIVKA-II, and Genetic Mutations
Source: Medicina (Kaunas). 2026 Mar 10;62(3):508. doi: 10.3390/medicina62030508 (PMC13028399; doi:10.3390/medicina62030508)
Supplement: Supplementary file 1 [file medicina-62-00508-s001.zip › medicina-4123327-supplementary.pdf]

**Supplementary Table S1. List of targeted genes**

| | | | | | |
|---|---|---|---|---|---|
| ABL1 | CDK4 | FBXW7 | IDH2 | NOTCH1 | RAD54L |
| AKT1 | CDK6 | FGF1 | INPP4B | NOTCH2 | RAF1 |
| AKT2 | CDKN2A | FGF10 | JAK2 | NOTCH3 | RB1 |
| AKT3 | CEBPA | FGF14 | JAK3 | NPM1 | RET |
| ALK | CHEK1 | FGF19 | KDR | NRAS | RICTOR |
| APC | CHEK2 | FGF2 | KIF5B | NRG1 | ROS1 |
| AR | CREBBP | FGF23 | KIT | NTRK1 | RPS6KB1 |
| ARID1A | CSF1R | FGF3 | KMT2A | NTRK2 | SLX4 |
| ATM | CTNNB1 | FGF4 | KRAS | NTRK3 | SMAD4 |
| ATR | DDR2 | FGF5 | LAMP1 | PALB2 | SMARCB1 |
| AXL | DNMT3A | FGF6 | MAP2K1 | PAX3 | SMO |
| BAP1 | EGFR | FGF7 | MAP2K2 | PAX7 | SRC |
| BARD1 | EML4 | FGF8 | MCL1 | PDGFRA | STK11 |
| BCL2 | EP300 | FGF9 | MDM2 | PDGFRB | TERT |
| BCL6 | ERBB2 | FGFR1 | MDM4 | PIK3CA | TET2 |
| BRAF | ERBB3 | FGFR2 | MET | PIK3CB | TFRC |
| BRCA1 | ERBB4 | FGFR3 | MLH1 | PIK3CD | TMPRSS2 |
| BRCA2 | ERCC1 | FGFR4 | MLLT3 | PIK3CG | TP53 |
| BRIP1 | ERCC2 | FLI1 | MPL | PIK3R1 | TSC1 |
| BTK | ERG | FLT1 | MSH2 | PMS2 | TSC2 |
| CARD11 | ESR1 | FLT3 | MSH3 | PPARG | VHL |
| CCND1 | ETS1 | FOXL2 | MSH6 | PPP2R2A | XRCC2 |
| CCND2 | ETV1 | GEN1 | MTOR | PTCH1 | |
| CCND3 | ETV4 | GNA11 | MUTYH | PTEN | |
| CCNE1 | ETV5 | GNAQ | MYC | PTPN11 | |
| CD79A | EWSR1 | GNAS | MYCN | RAD51 | |
| CD79B | EZH2 | HNF1A | MYD88 | RAD51B | |
| CDH1 | FANCI | HRAS | NBN | RAD51C | |
| CDK12 | FANCL | IDH1 | NF1 | RAD51D | |
